# Breeding of Fukumi Fiber, a new six-rowed waxy hull-less barley cultivar containing high levels of β-glucan with a proanthocyanidin-free gene

**DOI:** 10.1270/jsbbs.24080

**Published:** 2025-06-21

**Authors:** Asuka Takahashi, Toji Yoshioka, Takashi Yanagisawa, Takashi Nagamine, Tomohiko Sugita

**Affiliations:** 1 Western Region Agricultural Research Center, National Agriculture and Food Research Organization (WARC/NARO), 1-3-1 Senyu, Zentsuji, Kagawa 765-8508, Japan; 2 Institute of Crop Science, National Agriculture and Food Research Organization (NICS), 2-1-2 Kannondai, Tsukuba, Ibaraki 305-8518, Japan; 3 Bio-oriented Technology Research Advancement Institution, 16th floor, Parale Mitsui Building, 8 Higashida, Kawasaki, Kanagawa 210-0005, Japan; 4 Central Region Agricultural Research Center, National Agriculture and Food Research Organization (CARC/NARO), 1-2-1 Inada, Joetsu, Niigata 943-0193, Japan; 5 Western Region Agricultural Research Center, National Agriculture and Food Research Organization (WARC/NARO), 6-12-1 Nishifukatsu, Fukuyama, Hiroshima 721-8514, Japan

**Keywords:** hull-less barley, food, β-glucan, waxy, polyphenol, proanthocyanidin-free, discoloration

## Abstract

Fukumi Fiber, a new six-rowed hull-less barley cultivar, has an extremely high β-glucan content; this is the world’s first cultivar with two genes (*wax* and *amo1*) boosting the content of β-glucan and one gene (*ant28.2131*) suppressing the browning reaction after cooking, to our knowledge. The β-glucan content of pearled barley is 13.2% in Fukumi Fiber, and is approximately three times higher than that of the standard barley cultivar Ichibanboshi and approximately two times higher than that of the waxy cultivars Daishimochi and Kirari-mochi. Fukumi Fiber has a standard plump grain percentage required for a six-rowed hull-less barley cultivars. The yield is over 10% higher than that of Ichibanboshi. Fukumi Fiber is suitable for cultivation in the plains of central and western Japan and was released in 2018. It can be used for cooked pearled barley and various purposes such as confectionery, noodles, and bread. The spread of this cultivar is expected to lead to a stable supply and the expansion of high-value-added domestic waxy barley.

## Introduction

Barley grain is richer in 1-3,1-4 β-glucan (β-glucan), a water-soluble dietary fiber, than other grains such as rice and wheat. It suppresses elevated blood glucose levels (Yokoyama *et al.* 1997), reduces blood cholesterol (Ikegami *et al.* 1996, Oda *et al.* 1993), and improves immune function (Tada *et al.* 2008, Vetvicka *et al.* 1996). To label health claims on the products, food companies must prepare products that ensure a certain amount of β-glucan, and agricultural producers must produce barley raw materials that meet this requirement. Therefore, cultivars with high β-glucan content are important and in demand by consumers and producers.

Waxy barley has a sticky texture, a desirable trait for Japanese cooked pearled barley (Yanagisawa *et al.* 2011, Yoshikawa *et al.* 1998), and has higher β-glucan content than the normal barley (Ullrich *et al.* 1986). Barley has been used in Japanese food culture since ancient times, and various waxy cultivars have been selected and bred in Japan. Six-rowed hull-less cultivar Daishimochi, was bred by introducing low-amylose *wax* gene from waxy landrace Mochimugi D (Doi *et al.* 1999). The two-rowed hull-less cultivar Kirari-mochi is proanthocyanidin-free (*ant28.494*) as well as amylose-free with *wax* gene from Shikoku hadaka 97, and achieved higher β-glucan content and lower levels of discoloration of cooked pearled barley after incubation at higher temperatures than Ichibanboshi (a standard cultivar of hull-less barley) (Yanagisawa *et al.* 2011). β-glucan content became further higher by the six-rowed hulled cultivar Kihadamochi bred by introducing the amylose-free *wax* gene from Azhul, which has higher β-glucan content than other amylose-free cultivars such as Kirari-mochi (Tonooka *et al.* 2022). Incidentally, the alleles of the *wax* gene are known to be different among Mochimugi D, Shikoku hadaka 97, and Azhul (Hu *et al.* 2014). In these waxy cultivars Daishimochi, Kirari-mochi, and Kihadamochi, the β-glucan content of pearled barley is approximately 6%–8% (Doi *et al.* 1999, Tonooka *et al.* 2022, Yanagisawa *et al.* 2011). A much higher content (12.8%) of β-glucan was achieved by the two-rowed hulless cultivar Waxy Fiber, which has both *lys5h* and amylose-free (*wax*) (Yanagisawa *et al.* 2016). However, in the *lys5h* line, starch synthesis is considerably reduced because of the reduced ability of the amyloplast to incorporate the substrate ADP-glucose, which is the site of starch synthesis (Patron *et al.* 2004). Therefore, Waxy Fiber has shrunken grains, lower yields, poor plump grain percentage, and poor pearling quality (Yanagisawa *et al.* 2016). For such a reason, the cultivars bred and marketed to date have a β-glucan content of 6%–8%, and those with higher content have poor grain quality and low yields. Therefore, higher β-glucan cultivars that combine high yield, superior grain quality and stability are requisite.

To our knowledge, Fukumi Fiber is the world’s first cultivar having triple recessive alleles, *amo1* (high amylose), *wax*, and *ant28.2131*. Amylose and β-glucan content are known to be high in the *amo1* genotype (Fastnaught *et al.* 1996, Merritt 1967, Schondelmaier *et al.* 1992). Lines with both *amo1* and *wax* have even higher β-glucan content (Fujita *et al.* 1999). The β-glucan content of pearled barley is 13.2% in Fukumi Fiber. It is approximately three times as much as the standard cultivar Ichibanboshi and approximately twice as much as Daishimochi and Kirari-mochi.

Fukumi Fiber has the proanthocyanidin-free allele *ant28.2131*, which is resistant to browning after cooking like Kirari-mochi. The average yield is over 10% higher than that of Ichibanboshi. Boosting the production of this cultivar is expected to lead to a stable supply and the expansion of high-value-added domestic waxy barley.

## Materials and Methods

### Breeding process

**Fig. 1.**
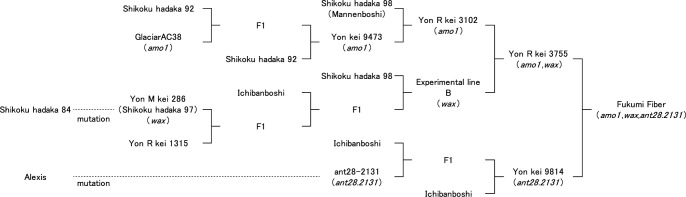
Pedigree of Fukumi Fiber.

Fukumi Fiber was derived from a cross between Yon R Kei 3755 (*amo1*, *wax*) and Yon Kei 9814 (*ant28.2131*), and selected by pedigree method of breeding (Fig. 1). The *amo1* phenotype was identified by its high grain hardness. It was later verified using the starch synthase IIIa (*ssIIIa*) marker which was tightly linked to *amo1* locus (Li *et al.* 2011). Yon R Kei 3755 was crossed with Yon Kei 9814 as a male parent in 2009. Twenty-eight F_1_ plants were grown in a greenhouse, and 467 F_2_ plants were grown in the field in 2009. Sixteen F_3_ individuals with proanthocyanidin-free, waxy and high level of grain hardness were selected in 2011. Yield trials were initiated at the Western Region Agricultural Research Center, National Agriculture and Food Research Organization (WARC/NARO) in 2011. Local adaptability tests were conducted at two different stations in 2014. Performance tests for recommended cultivars were conducted at a total of eight different prefectural stations from 2016 to 2017, with some locations tested in multiple years. Fukumi Fiber was released in Japan in 2018.

### Plant material for yield trials

All the cultivars were sown in late November at Zentsuji, Kagawa, and grown using standard yield trial methods at WARC/NARO (Table 1). Ichibanboshi (Ito *et al.* 1995) was a standard six-rowed, uzu-type, hull-less cultivar in Japan. Daishimochi (Doi *et al.* 1999), Kirari-mochi (Yanagisawa *et al.* 2011), and Waxy Fiber (Yanagisawa *et al.* 2016) were used as reference cultivars of waxy barley. The percentage of grains greater than 2.2 mm for the two-row barley and 2.0 mm for the six-row barley was used as the plump grain percentage. Harvested grains had no sprouting damage.

### PCR amplification and restriction enzyme digestion of the *ssIIIa* gene

To identify *ssIIIa* variations, we performed Cleaved Amplified Polymorphic Sequence (CAPS) analysis (Li *et al.* 2011). PCR was performed using total DNA from leaves or seeds on a TaKaRa PCR Thermal Cycler Dice (Takara TP600), using the specific primer sets SSIIIa-P5F and SSIIIa-P5R designed by Li *et al.* (2011).

### Measurement of β-glucan content and dietary fiber

Total β-glucan content in pearled barley was analyzed according to McCleary and Glennie-Holmes (1985) using the β-Glucan assay kit (Megazyme Ltd., Ireland) and expressed on a dry weight basis. Dietary fiber content was determined using the modified Prosky method (Enzymatic - LC method) by Japan Food Research Laboratories.

### Measurement of pearling quality parameters

The grain hardness index (HI) of 100 whole grains was measured using the single-kernel characterization system 4100 (SKCS, Perten Instruments). The pearling time was measured as the time required to grind 200 g of grains to obtain a 60% pearl yield using the TM-05 grain-testing mill (Satake CO.). The pearling machine of the same type was replaced in 2015, therefore the pearling time values differ between the 2012–2014 and 2015–2021 growing years. In addition, pearled barley was autoclaved (105°C × 10 min), steamed for 40 min while reducing pressure, and measured the hue color immediately after cooking using a spectrophotometer (Minolta CM-3500d). After measurement, the cooked pearled barley was incubated at 70°C for 18 h and measured the hue color in the same manner. Discoloration values reflected the change in pearled barley color after incubation.

## Results

### Agronomic characteristics

The agronomic characteristics of Fukumi Fiber are shown in Table 1. Fukumi Fiber has a slightly longer culm, slightly longer spike, and more spikes than Ichibanboshi, though the difference is statistically insignificant (Table 1). Fukumi Fiber headed one day after Ichibanboshi and matured three days later. The thousand-grain weight was lower than that of Ichibanboshi but higher than that of Daishimochi, although the difference was statistically insignificant. The plump grain percentage was above 95%, although it was lower than that of Ichibanboshi. The yield was higher by 13.7%, 26.7%, and 19.0% compared with Ichibanboshi, Dishimochi, and Kirari-mochi, respectively.

Fukumi Fiber was resistant to Barley yellow mosaic virus (BaYMV) and Japanese soil-borne wheat mosaic virus (JSBWMV), and its resistance to powdery mildew and scab was moderate, the same as that of Ichibanboshi (Table 2). Fukumi Fiber was more susceptible to pre-harvest sprouting than Ichibanboshi and Daishimochi but was similar to Kirari-mochi, which is also proanthocyanidin-free.

**Fig. 2.**
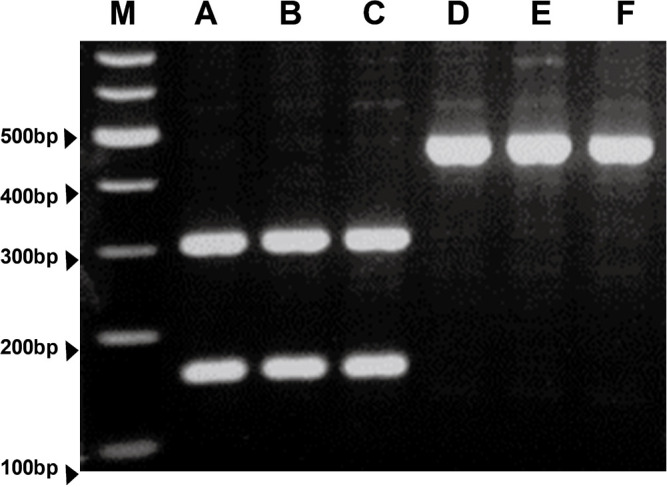
CAPS analysis of *ssIIIa* marker to estimate *amo1* genotype. M; molecular marker, Lane A; Fukumi Fiber (*amo1*), Lane B; EL-A (*amo1*), Lane C; Glacier AC38 (*amo1*), Lane D; Ichibanboshi, Lane E; Daishimochi, Lane F; Kirari-mochi.

**Fig. 3.**
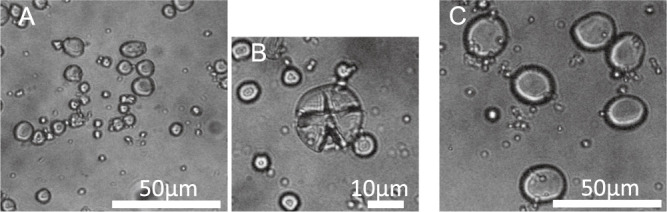
Small and cracked starch granules of Fukumi Fiber. A and B; Fukumi Fiber, C; Ichibanboshi. Images of starch granules were obtained with a Carl Zeiss Axiophot optical microscope at magnifications of 20 (A, C) and 40 (B).

The *amo1* genotype was first estimated by grain hardness and later verified by PCR analysis of the *ssIIIa* marker (Fig. 2). Genetic mapping studies have indicated that *ssIIIa* is very tightly linked to the *amo1* locus (Li *et al.* 2011). Lines with *amo1* can be selected using the *ssIIIa* marker (Li *et al.* 2011). Glacier AC38 is a mutant with a high amylose (*amo1*) gene derived from Glacier (Merritt 1967). Experimental line A (EL-A) with the wild-type *Wax* allele (normal amylose) and *amo1* genes was derived from the Ichibanboshi/Yon R Kei 3102 (*amo1*) cross. When examined using CAPS analysis of the *ssIIIa* marker, the cultivars with wild-type alleles of *ssIIIa*, Ichibanboshi, Daishimochi, and Kirari-mochi, showed a fragment of 464 bp. In contrast, the cultivars/lines with *amo1*, Fukumi Fiber, EL-A, and GlacierAC38 showed DNA fragments of 303 and 161 bp, respectively. The starch granules of Fukumi Fiber were smaller and more cracked than those of Ichibanboshi (Fig. 3).

### Pearling characteristics and β-glucan contents

Quality parameters, β-glucan content, and dietary fiber of pearled barley are listed in Table 3. Fukumi Fiber had a higher SKCS HI and a longer milling time than other cultivars. The broken kernel rate of Fukumi Fiber was significantly lower than that of Ichibanboshi, and was also lower than that of Daishimochi and Kirari-mochi, although the difference was not significant. The β-glucan content of the pearled barley of Fukumi Fiber is approximately three times higher than that of Ichibanboshi and approximately two times higher than that of Daishimochi and Kirari-mochi. The soluble and insoluble fiber contents of pearled barley in Fukumi Fiber were higher than in other cultivars.

**Fig. 4.**
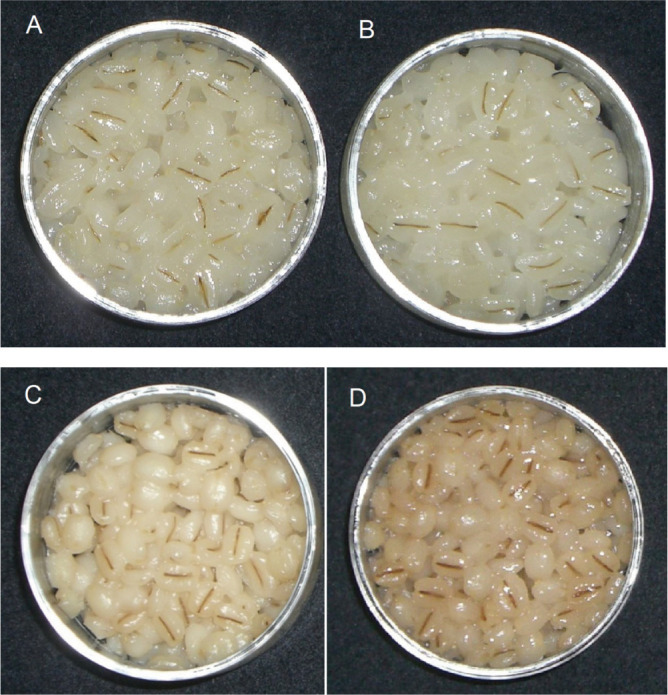
Cooked pearled barley incubated at 70°C for 18 h. A; Fukumi Fiber, B; Kirari-mochi, C; Ichibanboshi, D; Daishimochi.

The color parameters of the cooked pearled barley of Fukumi Fiber are shown in Table 3. The L* value of cooked pearled Fukumi Fiber grains was as high as that of Ichibanboshi and Kirari-mochi. Discoloration in L* and a* of cooked pearled barley after incubation at 70°C was suppressed in Fukumi Fiber compared to those in Ichibanboshi and Daishimochi. Like Kirari-mochi, Fukumi Fiber pearled barley hardly turned brown even after cooking and incubation at 70°C (Fig. 4).

### Comparison between Fukumi Fiber and other high β-glucan cultivar

**Fig. 5.**
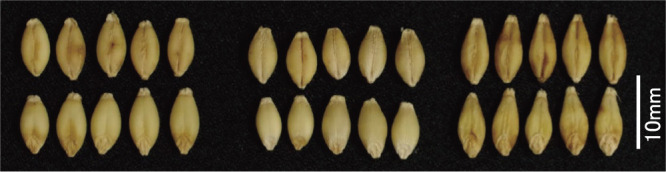
Grains. Fukumi Fiber grains do not have a shrunken surface unlike Waxy Fiber. From left, Fukumi Fiber, Ichibanboshi, and Waxy Fiber.

**Fig. 6.**
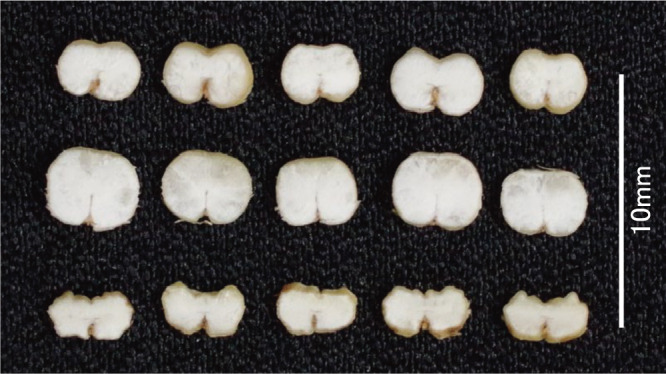
Cross section of grains. Grain plumpness of Fukumi Fiber is intermediate between Ichibanboshi and Waxy Fiber with *lys5h*. From above, Fukumi Fiber, Ichibanboshi, and Waxy Fiber.

The agronomic characteristics and pearling quality parameters are listed in Tables 4 and 5. Fukumi Fiber had the highest β-glucan content of the pearled barley among the cultivars examined, being similar to Waxy Fiber with both *lys5h* and *wax*. The yield of Fukumi Fiber was 32.1% higher than that of Waxy Fiber. The percentage of plump grain of Fukumi Fiber was not significantly different from that of the standard cultivar, but that of Waxy Fiber with *lys5h* was significantly lower, with the shrunk grains (Figs. 5, 6, Table 4). Fukumi Fiber has less seed plumpness than Ichibanboshi, but more than Waxy Fiber (Fig. 6). Fukumi Fiber had the highest SKCS HI among the cultivars examined; it also had a longer pearling time, and fewer broken kernels than the other cultivars. The L* values of cooked pearled barley were higher than those of Waxy Fiber. The discoloration evaluated by changes in L* and a* of cooked pearled barley was limited in Fukumi Fiber compared to those in Waxy Fiber.

## Discussion

### Agronomic characteristics

Previous studies have shown that two genes *wax* and *amo1* interact and further increase the β-glucan content (Fujita *et al.* 1999). Grains of lines with *wax* and *amo1* do not shrink unlike Waxy Fiber, which is a high β-glucan cultivar having *wax* and *lys5h*. However, the grain weight is often inferior due to low starch content and poor filling in the lines with *wax* and *amo1*. Of such lines, Yon kei 9519 has an inferior yield and a low percentage of plump grain (Tonooka *et al.* 2018). Fukumi Fiber is a cultivar that has been selected to address these shortcomings. Its *amo1* genotype was verified using DNA marker analysis (Fig. 2) and cracks of starch granules (Fig. 3). The starch from the *amo1* endosperm has a high amylose content and exhibits a prominent degree of cracking at the surface of the granules (Boren *et al.* 2008). This cultivar has no major drawbacks in yield and grain quality compared to the standard barley cultivars (Tables 1–5). The yield of Fukumi Fiber was over 10% higher than that of Ichibanboshi (Tables 1, 4). Although there was no significant difference in yield-related traits, Fukumi Fiber has a slightly longer culm, slightly longer spike, and more spikes than Ichibanboshi, suggesting its higher yield potential. The plump grain percentage of Fukumi Fiber was above 94% (Tables 1, 4) and met the Grading Standards for Agricultural Products. It was lower than that of the standard cultivar Ichibanboshi, but higher than that of the high-β-glucan-content cultivar Waxy Fiber. Fukumi Fiber has a grain-filling period 2–4 days longer than Ichibanboshi and 2–3 days longer than Daishimochi (Tables 1, 4). Because of the long grain-filling period, it is considered that the grains were full even though the rate of starch synthesis was slow. If ultra-high β-glucan and plump grains are required, lengthening the grain-filling period and increasing photosynthetic capacity are conceivable.

### Quality characteristics

Fukumi Fiber had the highest β-glucan content of the pearled barley among the cultivars examined; it also had the highest SKCS HI. Henry and Cowe (1990) reported a positive relationship between the β-glucan content and grain hardness. We examined the correlation between β-glucan content and pearling characteristics in the cultivars tested. For the cultivars shown in Table 3, the correlation coefficients between β-glucan content and SKCS HI and pearling time were 0.986 and 0.997, respectively, both statistically significant at the 5% and 1% levels. In contrast, the correlation with the broken kernel rate was not significant but showed a coefficient of –0.703. Furthermore, no correlations were significant for the cultivars shown in Table 5. However, the coefficients for SKCS HI, pearling time, and broken kernel rate were relatively high at 0.869, 0.877, and –0.706, respectively. Therefore, it suggested that the higher β-glucan content of Fukumi Fiber resulted in a higher SKCS HI and longer pearling time.

The browning of cooked pearled barley grains is significantly correlated with the content of flavanol, which is the major factor for the browning (Kohyama *et al.* 2010). The main flavanols quantified in the analyzed barley varieties were catechin, two dimers (procyanidin B3 and prodelphinidin B3), and four trimers (Holtekjølen *et al.* 2006). Fukumi Fiber is proanthocyanidin-free, resulting in a brighter color and less browning of cooked pearled barley. Proanthocyanidin-free cultivars such as Kirari-mochi show poor tolerance to pre-harvest sprouting (Nagamine *et al.* 2006, Yanagisawa *et al.* 2011). Himi *et al.* (2012) reported that *ant28* encodes Hvmyb10, an R2R3 MYB domain protein that regulates proanthocyanidin accumulation in developing grains. The *ant28-494* allele contains a nucleotide change predicted to create a stop codon, while the *ant28-2131* allele has a change which likely leads to the loss of five amino acids (Himi *et al.* 2012). The germination rate was slightly lower in Fukumi Fiber with *ant28-2131* than in Kirari-mochi with *ant28-494*; however, no significant difference was observed (Table 2). In addition, genes such as *Qsd1* and *Qsd2* are known to be involved in pre-harvest sprouting tolerance (Nakamura *et al.* 2016, Sato *et al.* 2016). Taira *et al.* (2021) suggested that the improvement in pre-harvest sprouting tolerance of Shirayuri Nijo, compared to Shiratae Nijo, in *ant28-494* can be attributed to the dormancy-type variation of *Qsd1* and the introduction of the genetic background of Haruka Nijo, which has strong dormancy. Fukumi Fiber had the dormancy-type *Qsd1* and *Qsd2* alleles (Table 2). Therefore, further improvement of pre-harvest sprouting tolerance in Fukumi Fiber may require the introduction of an *ant* gene with less impact on pre-harvest sprouting than *ant28* or introduction of novel sprouting tolerance factors that are distinct from *Qsd1* and *Qsd2*.

Fukumi Fiber is already grown commercially in western Japan and is used in cooked pearled barley and other products. Fukumi Fiber, with its extremely high β-glucan and good cooked pearled barley, has the potential to be used for conventional cooked pearled barley with rice, as well as for various other applications, such as for bread, noodles, cereal, and confectionery when ground into barley flour. The spread of this cultivar is expected to lead to a stable supply and the expansion of high-value-added domestic waxy barley.

## Author Contribution Statement

A.T., T.Yanagisawa and T.N. designed this study, and A.T., T.Yanagisawa, T.Yoshioka, T.N. and T.S. were involved in the breeding process, and A.T. and T.S. performed DNA marker identification, and A.T. wrote the paper.

## Figures and Tables

**Table 1.** Agronomic characteristics of Fukumi Fiber (Means of 2012–2017 growing years)

Cultivar	Row type	Semi-dwarf type	Waxy type	*amo1* type	Proantho-cyanidin-free type	Degree of spring habit*^a^*	Heading date	Maturity date	Culm length (cm)	Spike length (cm)	Number of spikes (/m^2^)	Grain yield		Volume weight (g/l)	1000-grain weight (g)	Plump grain (%)
												(kg/a)	(%)			
Fukumi Fiber	six	*uzu*	amylose-free	*amo1*	*ant28.2131*	V	5-Apr a*^b^*	18-May a	84 a	5.6 b	354 a	56.2 a	114	798 b	32.8 bc	95.9 b
Ichibanboshi	six	*uzu*	–	–	–	V	4-Apr a	15-May a	82 a	5.2 b	333 a	49.4 a	100	827 a	35.0 b	99.2 a
Daishimochi	six	*uzu*	Low-amylose	–	–	V	6-Apr a	17-May a	78 a	4.6 a	370 a	44.4 a	90	827 a	31.4 c	99.4 a
Kirari-mochi	two	–*^c^*	amylose-free	–	*ant28.494*	I	4-Apr a	17-May a	75 a	7.0 c	412 a	47.2 a	96	821 ab	41.4 a	98.8 a

Basel dressing N–P–K = 0.6–0.6–0.6 kg/a; top dressing N–K = 0.3–0.3 kg/a. The plot design was 4.95 m^2^ with two replications. *^a^* –: I: Spring type; V: Winter type. *^b^* The same letters in the same column are not significantly different (P < 0.05), as revealed by Tukey’s test. *^c^* –: Normal type.

**Table 2.** Evaluation data of resistance and tolerance

Cultivar	Disease resistance				Lodging resistance (culm breaking)*^e^*	Pre-harvest sprouting tolerance		*Qsd1* type*^g^*	*Qsd2* type*^h^*
	BaYMV (types I–III)*^a^*	JSBWMV*^b^*	Powdery mildew*^c^*	Scab*^d^*		Judgment*^e^*	Germination rate (%)*^f^*		
Fukumi Fiber	R	R	M	M	R	S	73.5 a*^i^*	D	D
Ichibanboshi	R	R	M	M	MR	R	5.8 b	D	D
Daishimochi	M*^j^*	n.t*^l^*	MS*^j^*	M*^j^*	R	R	5.8 b	n.t.	n.t.
Kirari-mochi	RR*^k^*	n.t.	RR*^k^*	MR*^k^*	R	S	80.8 a	D	D

RR: highly resistant, R: resistant, MR: moderately resistant, M: moderate, MS: moderately susceptible, S: susceptible, SS: highly susceptible. *^a^* Estimated in 2014–2017 growing years, tested in Tsukuba and Tsukubamirai, Ibaraki; Otsu, Kumamoto; and Tochigi, Tochigi. *^b^* Estimated in 2015–2016 growing years, tested in Tsukubamirai, Ibaraki. *^c^* Estimated in 2014, 2016, and 2017 growing years, tested in Tsukuba, Ibaraki. *^d^* Estimated in 2014–2017 growing years, tested in Tsukuba, Ibaraki; Tikugo and Tikushino, Fukuoka. *^e^* Estimated in 2012–2017 growing years, tested in Zuntsuji, Kagawa. *^f^* Means of 2012–2017 growing years. Germination ratio of 100 seeds of 18℃ on the 7th day of incubation, tested in Zuntsuji, Kagawa. *^g^* Determined using a marker based on the SNP in Exon 9 reported by Sato *et al.* (2016), with marker primers dCAPS-QSD1E9-TaqI-F: CCGTACAATCTTAGCGAGGATGGTGATTGGGGGCTTGAGATTTTGGAAGT and dCAPS-QSD1E9-TaqI-R: CTTTCCGACAAAATTCTACTATCTCCTCCT and restriction enzyme TaqI. D: dormancy allele. *^h^* Determined using a marker based on the SNP in Exon 7 reported by Nakamura *et al.* (2016), with marker primers CAPS-QSD2E7-2733RsaI-F: TGCATGAGGCAAGACACCTA and CAPS-QSD2E7-3158RsaI-R: TCACCTGCAGCATGAGATTG and restriction enzyme RsaI. D: dormancy allele. *^i^* The same letters in the same column are not significantly different (P < 0.05), as revealed by Tukey’s test. *^j^* Doi *et al.* 1999 *^k^* Yanagisawa *et al.* 2011 *^l^* n.t.: Not tested.

**Table 3.** Pearling quality parameters of Fukumi Fiber

Cultivar	SKCS Hardness index*^a^*	Pearling time*^a^* (s)	Whiteness of pearled grain*^a^* (%)	Broken kernel rate*^a^* (%)	β-glucan*^b^* (%)	Dietary fiber*^c^*				Cooked pearled barley*^b^*				Discoloration of the cooked pearled barley after incubating*^b^*		
						Soluble	Insoluble	Total		L*	a*	b*		L*	a*	b*
Fukumi Fiber	103 a*^d^*	1149 a	41.1 b	0.7 b	13.2 a	10.6	10.1	20.7		74.4 a	1.3 ab	17.6 a		–2.9 a	+0.9 b	–0.7 b
Ichibanboshi	51 c	422 b	45.3 a	28.1 a	4.3 c	3.9	4.4	8.3		74.4 a	1.1 ab	11.6 b		–7.7 b	+3.8 a	+3.2 a
Daishimochi	71 b	635 b	44.1 ab	4.1 b	6.3 b	n.t.*^e^*	n.t.	n.t.		71.4 a	1.7 a	12.3 b		–8.1 b	+3.7 a	+3.5 a
Kirari-mochi	68 b	652 b	45.9 a	6.7 b	6.6 b	6.7	2.9	9.5		73.8 a	0.8 b	16.3 a		–2.0 a	+1.0 b	–0.0 b

Basel dressing N–P–K = 0.6–0.6–0.6 kg/a; top dressing N–K = 0.3–0.3 kg/a. The plot design was 4.95 m^2^ with two replications. *^a^* Means of 2012–2017 growing years. *^b^* Means of 2013–2017 growing years. *^c^* Grew in 2014. *^d^* The same letters in the same column are not significantly different (P < 0.05), as revealed by Tukey’s test.

**Table 4.** Agronomic characteristics of high β-glucan cultivars/lines (Means of 2019–2021 growing years)

Cultivar	Heading date	Maturity date	Culm length (cm)	Spike length (cm)	Number of spikes (/m^2^)	Grain yield		Volume weight (g/l)	1000-grain weight (g)	Plump grain (%)
						(kg/a)	(%)			
Fukumi Fiber	27-Mar a*^a^*	13-May a	92 a	5.4 b	517 a	68.6 a	111	796 b	31.4 c	94.1 a
Waxy Fiber	25-Mar a	8-May a	85 a	7.4 a	568 a	51.9 a	84	747 c	35.6 b	51.0 b
Ichibanboshi	26-Mar a	8-May a	86 a	5.1 b	511 a	61.6 a	100	828 ab	33.8 bc	99.3 a
Daishimochi	29-Mar a	12-May a	84 a	4.4 b	465 a	64.2 a	104	838 a	32.2 c	99.6 a
Kirari-mochi	25-Mar a	12-May a	79 a	6.9 a	634 a	71.3 a	116	813 ab	40.1 a	98.6 a

Basel dressing N–P–K = 0.6–0.6–0.6 kg/a; top dressing N–K = 0.3–0.3 kg/a. The plot design was 3.15 m^2^ with two replications. *^a^* The same letters in the same column are not significantly different (P < 0.05), as revealed by Tukey’s test.

**Table 5.** Pearling quality parameters of high β-glucan cultivars/lines (Means of 2019–2021 growing years)

Cultivar	Row type	Semi-dwarf type	Waxy type	*amo1* type	*lys5h* type	Proantho-cyanidin-free type	SKCS Hardness index	Pearling time (s)	Whiteness of pearled grain (%)	Broken kernel rate (%)	β-glucan (%)	Cooked pearled barley				Discoloration of the cooked pearled barley after incubating		
												L*	a*	b*		L*	a*	b*
Fukumi Fiber	six	uzu	amylose-free	*amo1*	–	*ant28.2131*	110 a*^a^*	732 a	35.0 c	0.4 b	15.1 a	74.1 b	1.4 c	19.7 a		–3.7 a	+0.8 b	–1.2 b
Waxy Fiber	two	–*^b^*	amylose-free	–	*lys5h*	–	84 b	523 b	35.8 bc	1.0 b	14.3 a	67.6 c	2.8 a	18.5 a		–9.6 b	+3.8 a	+0.6 ab
Ichibanboshi	six	uzu	–	–	–	–	56 c	302 c	42.5 a	24.1 a	4.3 c	76.7 a	1.0 c	12.0 b		–7.4 b	+3.4 a	+2.3 a
Daishimochi	six	uzu	Low-amylose	–	–	–	79 b	473 b	40.6 ab	2.5 b	6.8 b	71.5 b	2.1 b	14.0 b		–7.9 b	+3.3 a	+2.3 a
Kirari-mochi	two	–	amylose-free	–	–	*ant28.494*	72 b	426 bc	41.7 a	5.4 b	6.8 b	72.8 b	1.1 c	17.5 a		–3.0 a	+0.8 b	–1.1 b

Basel dressing N–P–K = 0.6–0.6–0.6 kg/a; top dressing N–K = 0.3–0.3 kg/a. The plot design was 3.15 m^2^ with two replications. *^a^* The same letters in the same column are not significantly different (P < 0.05), as revealed by Tukey’s test. *^b^* –: Normal type.
